# Simvastatin and Benznidazole-Mediated Prevention of *Trypanosoma cruzi*-Induced Endothelial Activation: Role of 15-epi-lipoxin A4 in the Action of Simvastatin

**DOI:** 10.1371/journal.pntd.0003770

**Published:** 2015-05-15

**Authors:** Carolina Campos-Estrada, Ana Liempi, Fabiola González-Herrera, Michel Lapier, Ulrike Kemmerling, Barbara Pesce, Jorge Ferreira, Rodrigo López-Muñoz, Juan D. Maya

**Affiliations:** 1 Molecular and Clinical Pharmacology Program, Biomedical Sciences Institute (ICBM), Faculty of Medicine, University of Chile, Santiago, Chile; 2 Anatomy and Developmental Biology Program, Biomedical Sciences Institute (ICBM), Faculty of Medicine, University of Chile, Santiago, Chile; 3 Instituto de Farmacología y Morfofisiología, Facultad de Ciencias Veterinarias, Universidad Austral de Chile, Valdivia, Chile; Harvard School of Public Health, UNITED STATES

## Abstract

*Trypanosoma cruzi* is the causal agent of Chagas Disease that is endemic in Latin American, afflicting more than ten million people approximately. This disease has two phases, acute and chronic. The acute phase is often asymptomatic, but with time it progresses to the chronic phase, affecting the heart and gastrointestinal tract and can be lethal. Chronic Chagas cardiomyopathy involves an inflammatory vasculopathy. Endothelial activation during Chagas disease entails the expression of cell adhesion molecules such as E-selectin, vascular cell adhesion molecule-1 (VCAM-1) and intercellular cell adhesion molecule-1 (ICAM-1) through a mechanism involving NF-κB activation. Currently, specific trypanocidal therapy remains on benznidazole, although new triazole derivatives are promising. A novel strategy is proposed that aims at some pathophysiological processes to facilitate current antiparasitic therapy, decreasing treatment length or doses and slowing disease progress. Simvastatin has anti-inflammatory actions, including improvement of endothelial function, by inducing a novel pro-resolving lipid, the 5-lypoxygenase derivative 15-epi-lipoxin A4 (15-epi-LXA4), which belongs to aspirin-triggered lipoxins. Herein, we propose modifying endothelial activation with simvastatin or benznidazole and evaluate the pathways involved, including induction of 15-epi-LXA4. The effect of 5 μM simvastatin or 20 μM benznidazole upon endothelial activation was assessed in EA.hy926 or HUVEC cells, by E-selectin, ICAM-1 and VCAM-1 expression. 15-epi-LXA4 production and the relationship of both drugs with the NFκB pathway, as measured by IKK-IKB phosphorylation and nuclear migration of p65 protein was also assayed. Both drugs were administered to cell cultures 16 hours before the infection with *T*. *cruzi* parasites. Indeed, 5 μM simvastatin as well as 20 μM benznidazole prevented the increase in E-selectin, ICAM-1 and VCAM-1 expression in *T*. *cruzi*-infected endothelial cells by decreasing the NF-κB pathway. In conclusion, Simvastatin and benznidazole prevent endothelial activation induced by *T*. *cruzi* infection, and the effect of simvastatin is mediated by the inhibition of the NFκB pathway by inducing 15-epi-LXA4 production.

## Author Summary

Chagas disease, caused by the protozoan *Trypanosoma cruzi*, affects more than 10 million people in Latin America. In the chronic phase, a lethal complication may develop: Chronic Chagasic Cardiomyopathy. In this condition the vascular lining, the endothelium, is involved and participates in disease progression. Benznidazole is the current treatment for Chagas disease. However, other useful drugs could be added to Chagas disease chemotherapy to improve associated processes such as endothelial dysfunction, thus decreasing length and adverse events of the conventional therapy. Simvastatin, a drug that decreases blood cholesterol, also has anti-inflammatory effects and improves endothelial function. Thus, we studied the effect of simvastatin and benznidazole on endothelial cells and their relation with the production 15-epi-lipoxin A4, an anti-inflammatory molecule. We found that simvastatin and benznidazole decreased endothelial activation since they reduced the adhesion of inflammatory cells. Simvastatin and benznidazole inhibited NFκB pathway, which is pro-inflammatory and in the case of simvastatin, this effect was mediated by the production of 15-epi-lipoxin A4. Thus, we provide the bases that support the future use of simvastatin in the treatment of cardiac Chagas disease.

## Introduction

Chagas disease (CD) afflicts more than ten million people in Latin-America, where it is endemic, and worldwide as a consequence of migration [1]. This disease is caused by *Trypanosoma cruzi*, a vector-borne flagellate protozoan that infects virtually any nucleated cell in its mammalian hosts [2]. CD evolves from an acute, frequently asymptomatic, unspecific phase towards a chronic, silent phase. In total, 30% of chronically infected patients develop clinical manifestations due to gastrointestinal or cardiac involvement. Finally, patients die because of cardiovascular complications such as heart failure, arrhythmias, or thromboembolism secondary to ventricular aneurysms. Indeed, chronic Chagas cardiomyopathy (CCC) is responsible for the high burden of disease and explains its high mortality [3]. CCC pathophysiology involves parasite permanence in myocardial tissue and persistence of immune system activation including generation of autoantibodies against cardiac cholinergic receptors and ultimately microvascular damage [4].

*T*. *cruzi* reportedly induces endothelial activation [5] as revealed by an increase in the expression of endothelial cell adhesion molecules (ECAMs) such as E-Selectin, vascular cell adhesion molecule-1 (VCAM-1) and intercellular cell adhesion molecule-1 (ICAM-1) [6] through a mechanism involving NF-κB activation [7]. Endothelial activation induces vasoconstriction, inflammatory cell recruitment favoring immune cell homing, and generation of a procoagulant environment that promotes local ischemia [8,9].

Current drug therapy is not one hundred percent curative, especially during the chronic phase, and has diverse adverse events that affect patient compliance and often require treatment suspension. Nonetheless, current advances in trypanocidal therapy have not generated drugs that exceed the effectiveness of current medications, although several triazole derivatives are promising [4]. Thus, a novel strategy is proposed that aims at some pathophysiological processes to facilitate current antiparasitic therapy, decreasing treatment length or doses and slowing disease progress. Previously, it was suggested that aspirin, a well-known and widely used medication, could perform this function [10]. Herein, we present evidence that statins, mainly simvastatin, can play a similar role. This drug decreases inflammatory infiltration in the hearts of *T*. *cruzi*-infected dogs [11]. This anti-inflammatory effect is part of the pleiotropic effects of statins, which has been related to a novel pro-resolving lipid that is an aspirin-triggered lipoxin, 15-epi-lipoxin A4 (15-epi-LXA4) [12].

The present report provides evidence that links the effects of simvastatin and benznidazole to *T*. *cruzi*-infection induced endothelial activation and the relationship between endothelial activation and 15-epi-LXA4 production. These two drugs decreased CAM expression and leukocyte adhesion in an *in vitro* infection model. Furthermore, the effect of benznidazole on endothelial activation is independent of the parasite, suggesting an independent anti-inflammatory action.

## Methods

### Cells

EA.hy926 cells (ATCC CRL2922) are a human umbilical vein cell line established by fusing primary human umbilical vein cells with a thioguanine-resistant clone of A549 by exposure to polyethylene glycol (PEG). Hybrid clones were selected in HAT medium and screened for factor VIII-related antigen. The cell line was cultured following reported conditions [13]. Cells were cultured on Iscove's Modified Dulbecco's Medium (IMDM, Biological Industries, Israel) supplemented with 10% v/v FBS, 100 U/mL penicillin, and 100 mg/mL streptomycin at 37°C and 5% CO_2_.

HL-60 cells (ATCC CCL240) are a promyelocytic cell line that was derived by S.J. Collins et al [14]. Peripheral blood leukocytes were obtained by leukapheresis from a 36-year-old Caucasian female with acute promyelocytic leukemia. The cell line was cultured with Iscove's Modified Dulbecco's Medium plus 10% v/v FBS.

HUVECs (C-015-10C, Cascade Biologics, Life Technologies, USA) are primary human umbilical vein endothelial cells that are pooled from multiple donors. Cells were cultured in medium 200 (Cascade Biologics, USA) that had been supplemented with low serum growth supplement (LSGS, Cascade Biologics).

### Parasites

*T*. *cruzi* trypomastigotes (Dm28c clone [15]) from our collection, were obtained from infected EA.hy926 cells. Cells were exposed to trypomastigotes (Dm28c clone) at a multiplicity of infection (MOI) of 5. Trypomastigotes were allowed to infect cells for 24 hours, after which the supernatant was removed and fresh medium was added. Trypomastigotes were released from EA.hy926 cells after four days of infection. The parasites were harvested and collected for viability assays and further cell infections.

### Drugs

Simvastatin, benznidazole, AA-861 (2-(12-hydroxydodeca-5,10-diynyl)-3,5,6-trimethyl-p-benzoquinone) were obtained from Sigma-Aldrich, USA. 15-epi 15-epi-lipoxin A4 (cat#90415), and 5(S),6(R)-Lipoxin A4 methylester (cat#10033) were from Cayman Chemical, USA. All drugs were dissolved in DMSO and controls were incubated with DMSO vehicle alone. DMSO final concentration at cell cultures was 0.025% v/v. For most of the experiments, simvastatin and benznidazol concentration was 5 and 20 μM, respectively. It has been reported that 5 μM simvastatin is effective in decreasing inflammation and expression of ECAMs [16] [17]. 20 μM benznidazole correspond to the IC_50_ for its trypanocidal action [18].

### Cell viability determination by tetrazolium reduction assay

The effect of the drug on all cells and parasite viability was evaluated through the tetrazolium salt (MTT, Sigma-Aldrich) reduction assay as described [19]. Drugs at concentrations ranging from 1 to 20 μM, dissolved in DMSO at a 0.025% v/v final concentration, were applied to 2.5x10^5^ cells/mL or 10^6^ parasite/mL culture medium. Cultures were incubated for 24 hours before adding MTT for 4 hours. The plates were incubated overnight with 10% SDS w/v in 0.01 M HCl at 37°C, and optical density (OD) was determined using a microplate reader (Labsystems Multiskan MS, Finland) at 570 nm. Under these conditions, the OD was directly proportional to viable cell number per well. All of the experiments were performed at least three times, and the data are shown as the means and their standard deviations from triplicate cultures.

### Expression of endothelial cell adhesion molecules by flow cytometry

5×10^5^ EA.hy926 or HUVEC cells/well were seeded in 6-well plates and exposed to various simvastatin and/or benznidazole concentrations for 24 hours. Then, cells were infected with trypomastigotes at a MOI of 10 and incubated for 16 hours. Cells were detached with 1X EDTA-PBS at 0.5 mM. The harvested cells were washed with cold PBS and centrifuged at 800 x g for 5 minutes. Then, cells were washed with flow cytometry buffer three times. 100 μL of cell suspensions were incubated for 45 min at 4°C in the dark with mouse monoclonal anti- human E-selectin (5 μL undiluted), ICAM-1(3 μL undiluted), and VCAM-1 (1 μL undiluted) that were conjugated with PE, FITC and APC, respectively (BioLegend, USA). Cell suspensions were analyzed by flow cytometry using a FACSAria-III flow cytometer (BD Biosciences, USA). A homogeneous cell population was selected by size vs. granularity in log scale for these two conditions.

### Immunofluorescence staining

2x10^4^ EA.hy926 cells/slide were seeded in Lab-Tek II Chamber Slide^TM^ (ThermoScientific, USA) and allowed to adhere overnight. Then, cells were incubated with 5 μM simvastatin or 20 μM benznidazole for 24 hours prior to *T*. *cruzi*- infection (Dm28c clone) at a MOI of 10. After 16 hours of infection, cells were fixed in 4% formaldehyde–0.1 M phosphate buffer (pH 7.3) for 10 min. Cells were blocked with 3% bovine serum albumin for 1 hour. Then, cells were incubated with monoclonal antibodies against E-selectin (1:100), VCAM-1 (1:250) and ICAM-1 (1:100) (from Abcam, UK) overnight at 4°C. The samples were washed with PBS and incubated with anti-rabbit IgG that had been conjugated with fluorescein (1:100) from Sigma-Aldrich for 1 h. Finally, nuclei were stained with DAPI for 5 minutes and mounted with Dako Fluorescence Mounting (Dako, USA). The cells were photographed using a Nikon Eclipse 400 fluorescence microscope (Nikon, Japan), and images were analyzed by mean intensity using ImageJ software (ImageJ 1.47v).

### Western blot

5x10^5^ endothelial cells were rinsed once with PBS and scraped. Proteins were extracted into 100 μL RIPA buffer plus a protease and phosphatase inhibitor cocktail (Radio-Immunoprecipitation Assay; Millipore Corporation, USA) at 4°C. Total protein was quantified with the Lowry assay. Samples in loading buffer (10% SDS, 50% glycerol, 0.5 M Tris, 0.1% bromophenol blue, and 1 M dithiothreitol, pH 6.8) were electrophoretically separated by 10% SDS-PAGE and transferred to a nitrocellulose membrane. Nitrocellulose membranes were incubated with blocking solution (5% w/v nonfat dry milk, 0.1% Tween-20 in Tris-buffered saline) at room temperature for 1 hour. Then, the membranes were incubated at 4°C overnight with 1 mL solution of primary antibodies against E-selectin (1:1000) and α-Tubulin (1:10.000) from Sigma-Aldrich, ICAM-1 (1:2000) and VCAM-1 (1:1000) from Abcam, 7 ml solution of p-IKK (1:1000), IKK (1:1000), p-IκB (1:1000) and IκB (1:1000) from Cell signaling (Cell Signaling Technologies, USA). Bound antibodies were detected with horseradish peroxidase-conjugated secondary antibodies and visualized by Lumminata forte (Millipore). The nitrocellulose was stripped between reprobes using Mild stripping (Millipore) for 10 minutes. Developed films were scanned, and band densitometry was analyzed using ImageJ software (ImageJ 1.47v).

### DAPI staining and intracellular amastigote quantification

2x10^4^ EA.hy926 cells/slide were seeded in Lab-Tek II Chamber Slides for 12 h. Cells were exposed to 5 μM simvastatin or 20 μM benznidazole for 24 hours prior to *T*. *cruzi*- infection (Dm28c clone) at a MOI of 10 for 16 hours. Then, the cells were washed and fixed in cold methanol (70%) overnight. The fixed cells were then washed, and 1 mL PBS (pH 7.4) was added. DNA was stained with DAPI (NucBlue; Molecular Probes, USA) following the manufacturer's instructions. The cells were photographed using a Nikon Eclipse 400 fluorescence microscope using 358 nm (excitation) and 461 nm (emission) wavelengths. In total, ten pictures were obtained per well, and each picture was counted using MATLAB software.

### Cytoskeletal staining

2x10^4^ EA.hy926 or HUVEC cells/slide cultures were seeded in Lab-Tek II Chamber Slides^TM^. Cells were treated with 1–20 μM simvastatin. After drug treatment, cells were fixed at room temperature for 10 min in 3.7% formaldehyde (v/v) in phosphate-buffered saline (PBS) for 10 minutes. Cytoskeleton was assessed using a F-Actin Visualization Biochem Kit (Cytoskeleton, USA), following manufacturer’s instructions. Briefly, cells were washed with cytoskeletal buffer, followed by permeabilization for 5 min with permeabilization buffer and blocking with serum-containing buffer (3% FBS in PBS with 0.02% sodium azide). The cells were incubated with tetrarhodamine isothiocyanate (TRITC)-phalloidin for 30 minutes to stain cytoskeletal F-actin. DNA was stained with DAPI (NucBlue; Molecular Probes, USA) following the manufacturer's instructions. The cells were photographed using a Nikon Eclipse 400 fluorescence microscope.

### Cell-cell adhesion assay

The leukocyte adhesion assay was performed with the Cytoselect Leukocyte-Endothelium Adhesion Assay kit (CBA-210; Cell Biolabs, USA). Briefly, EA.hy926 cells were cultured in 96-well plates that had been previously coated with gelatin for 1 hour. Confluent monolayers were treated with 1–10 μM simvastatin or 1–20 μM benznidazole for 24 hours followed by *T*. *cruzi* infection for 16 hours. LeukoTracker-labeled leukocytes (HL60 cells) were added to the monolayer and incubated for 90 min. After thorough washing, cells were lysed, and fluorescence was measured at 480 nm excitation/520 nm emission. The percentage of adherent leukocytes was calculated: % adherence = adherent signal/total signal. All of the determinations were performed in triplicate using a fluorescence microplate reader (Varioskan, Thermo Scientific).

### 15-epi-lipoxin A4 determination

8x10^5^ EA.hy926 cells/well were cultured in 6-well plates (IMDM 10% SFB). Then, the cells were treated with simvastatin 5 μM for 24 hours and infected at a MOI of 10 for 16 hours. 15-epi-LXA4 determination was performed by:

1. ELISA assay: the supernatant from the cell cultures was assayed using a 15-epi-LXA4 BioAssay ELISA Kit (USBiological, USA) following manufacturer’s instructions.
2. AxION DSA—TOF MS determination: To determine 15-epi-lipoxin A4 levels in EAhy926 cell culture supernatants, an AxION Direct Sample Analysis (DSA) that was integrated with an AxION 2 time-of-flight (TOF) mass spectrometer (PerkinElmer, Shelton, CT, USA) was employed. For extraction, all of the samples were loaded onto a solid phase extraction and eluted with 1 mL chloroform three times successively. Then, the samples were transferred into a clean tube, and the chloroform in the samples was evaporated in a water bath. Finally, the samples were resuspended in 100 μL ethanol, and 10 μL were pipetted onto a stainless mesh. For the calibration curve, 15-epi-lipoxin A4 (cat#90415, Cayman Chemical, USA) was diluted in PBS to achieve different final concentrations (see S1 Fig). From each tube, 10 μL was pipetted onto a stainless mesh, and DSA—TOF determination was performed. The AxION DSA conditions were as follows: 5 μA corona current, 250°C heater temperature, 80 psi auxiliary gas (N2) pressure, 4 L/min drying gas (N2) flow, and 25°C drying gas (N2) temperature. Run in negative ionization trap mode had a flight tube voltage of 10000 V. The capillary exit voltage was set to 155 V for normal MS analysis. Mass spectra were acquired with a mass range of 100–1000 m/z and an acquisition rate of 2 spectra/sec. To maintain mass accuracy, two lock mass ions were used (m/z 119.0400, m/z 556.0000 and m/z 805.9900). All of the samples were analyzed for only 10 sec. The standards used were 15-epi-lipoxin A4 (cat#90415, Cayman Chemical,) and 5(S),6(R)-Lipoxin A4 methyl ester (cat#10033, Cayman Chemical) as an internal standard. These were confirmed in seconds by accurate mass and isotopic distribution of parent and fragment ions using DSA/TOF and AxION software, and compound identification was performed using the standards. All of the samples were analyzed in triplicate.

### Confocal microscopy

2x10^4^ EA.hy926 cells were seeded in Lab-Tek II Chamber Slides^TM^ for 12 hours. Cells were fixed in 3.7% formaldehyde–0.1 M phosphate buffer (pH 7.3) for 10 min. They were then washed with cytoskeletal buffer followed by permeabilization for 5 min with Triton-X100 0.5%. Cells were blocked with 3% bovine serum albumin for 1 hour. Then, cells were incubated with monoclonal antibodies against p65 (1:100) from cell signaling overnight at 4°C. The samples were washed with PBS and incubated with anti-rabbit IgG conjugated with fluorescein (1:100) from Sigma-Aldrich for 1 hour. Finally, nuclei were stained with DAPI for 5 minutes and mounted in Dako fluorescence mounting media. The cells were photographed and ten pictures per well were obtained; images were analyzed using ImageJ software (ImageJ 1.47v).

### Statistical analysis

Statistical significance was established at p<0.05. The results represent the mean ± SD of triplicates. Normal data distribution was assessed using D'Agostino-Pearsons and Shapiro-Wilk analysis. One- and two-way ANOVA analysis (with Tukey’s or Bonferroni’s post-tests) was performed when required. All of the statistical analyses were performed using GraphPad Prism (5.0) software.

For the analysis of the effect of the combination of simvastatin and benznidazoles on ECAM expression, the combinatory index (CI) and isobolographic analysis was performed using CompuSyn software (ComboSyn, Inc. Paramus, NJ) in accordance with the Chou and Talalay’s principle [20]. The interaction between simvastatin and benznidazole, on E-Selectin, ICAM-1 and VCAM-1 was investigated by calculating the CI, where CI < 1, CI = 1 and CI > 1 indicate synergism, additive and antagonism, respectively.

## Results

### Simvastatin and benznidazole prevent the increase of *Trypanosoma cruzi* infection-induced cell adhesion molecule surface expression

**Fig 1:**
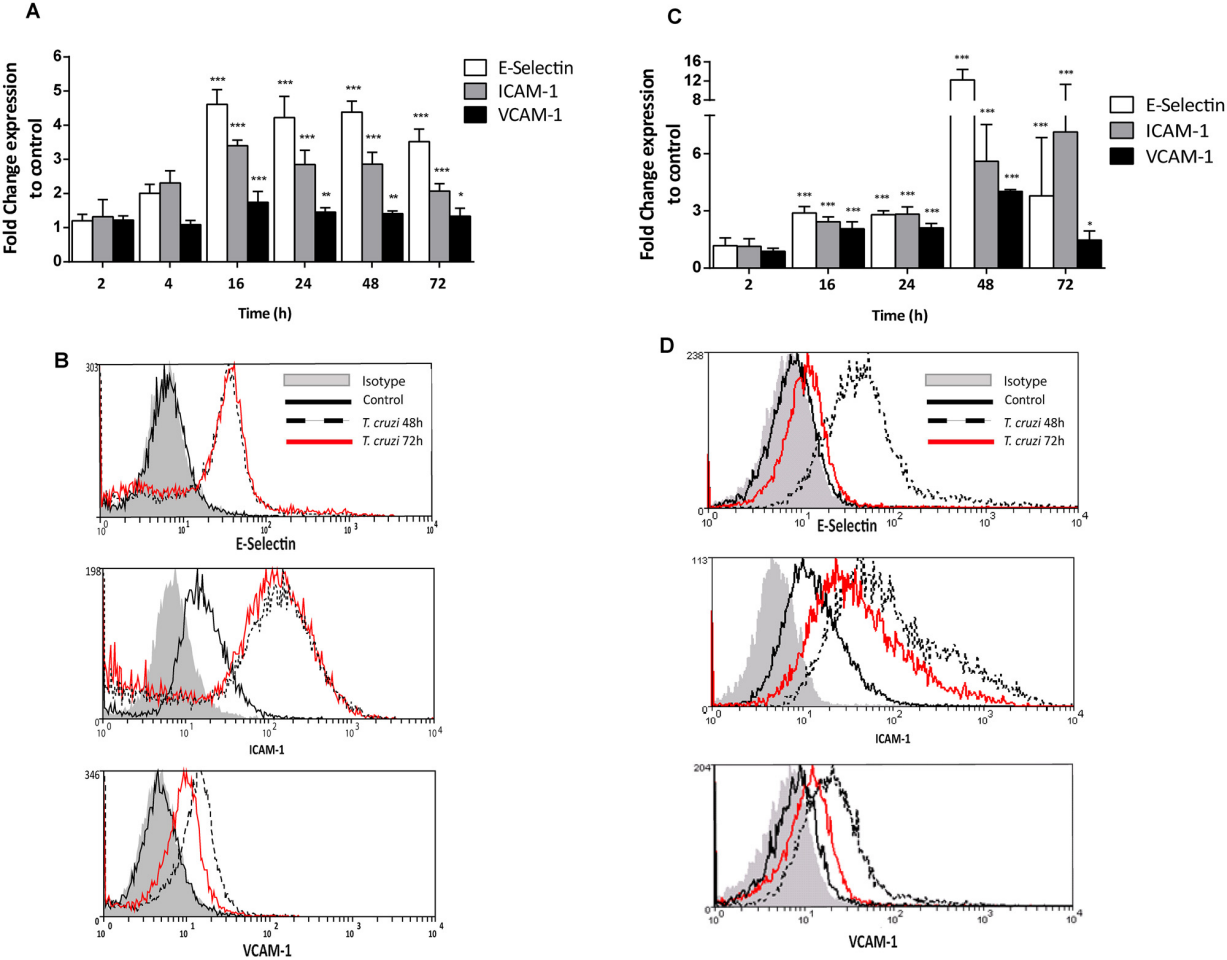
In *Trypanosoma cruzi-*infected endothelial cells, cell adhesion molecule surface expression is increased. Endothelial—like (EA.hy926) (A, B) and HUVECs (C, D) were infected with *T*. *cruzi* (Dm28c) trypomastigotes at a MOI of 10. E-Selectin, ICAM-1 and VCAM-1 expression was determined at the times indicated in the figures. Flow cytometry was performed using mouse IgG antibodies conjugated with PE, FITC-A, and APC against human E-Selectin, ICAM-1, and VCAM-1, respectively. Panels A and C represent mean fluorescence intensity (MFI) calculations, and panels B and D demonstrate representative histograms at 48 and 72 hours post-infection. Each group was compared with the respective IgG_1_ and IgG_2A_ isotype match and normalized. The data represent the mean ± SD of MFI values from three independent experiments. *** *p*<0.0001; **p<0.01; *p<0.5 vs. control using two-way ANOVA and Bonferroni post-test analysis.

To determine the optimal time point of maximal expression of ECAMs in *T*. *cruzi*-infected endothelial cells, a kinetic pattern of expression was determined by flow cytometry. Fig 1 demonstrates the mean fluorescence intensity (MFI) and representative histograms for each ECAM analyzed as obtained after 48 and 72 hours of *T*. *cruzi* infection. E-Selectin, ICAM-1 and VCAM-1 surface expression on endothelial-like EA.hy926 (Fig 1A and 1B) and HUVEC (Fig 1C and 1D) cells increased in a time-dependent manner. However, the expression behavior was slightly different from each cell model. In EA.hy926 cells, after reaching their maximum expression at 16 hours, a steady state was attained with a slow trend to decreased expression without reaching control values (Fig 1A). For HUVECs, there was a statistically significant increase at 16 hours for all three adhesion molecules (two-way Anova with bonferroni post-test, p<0.05). However, peak expression was reached at 48 hours and declined until 72 hours, and only a sustained ICAM-1 expression remained (Fig 1C). Constitutive ICAM-1 expression could likely account for this observation [21]. Indeed, as demonstrated in Fig 1C and 1D, there is a slight deviation in the ICAM-1 fluorescence signal in both HUVEC and EA.hy926 uninfected cells. As a consequence of these results, peak ECAM expression was set at 16 hours for further assays.

**Fig 2:**
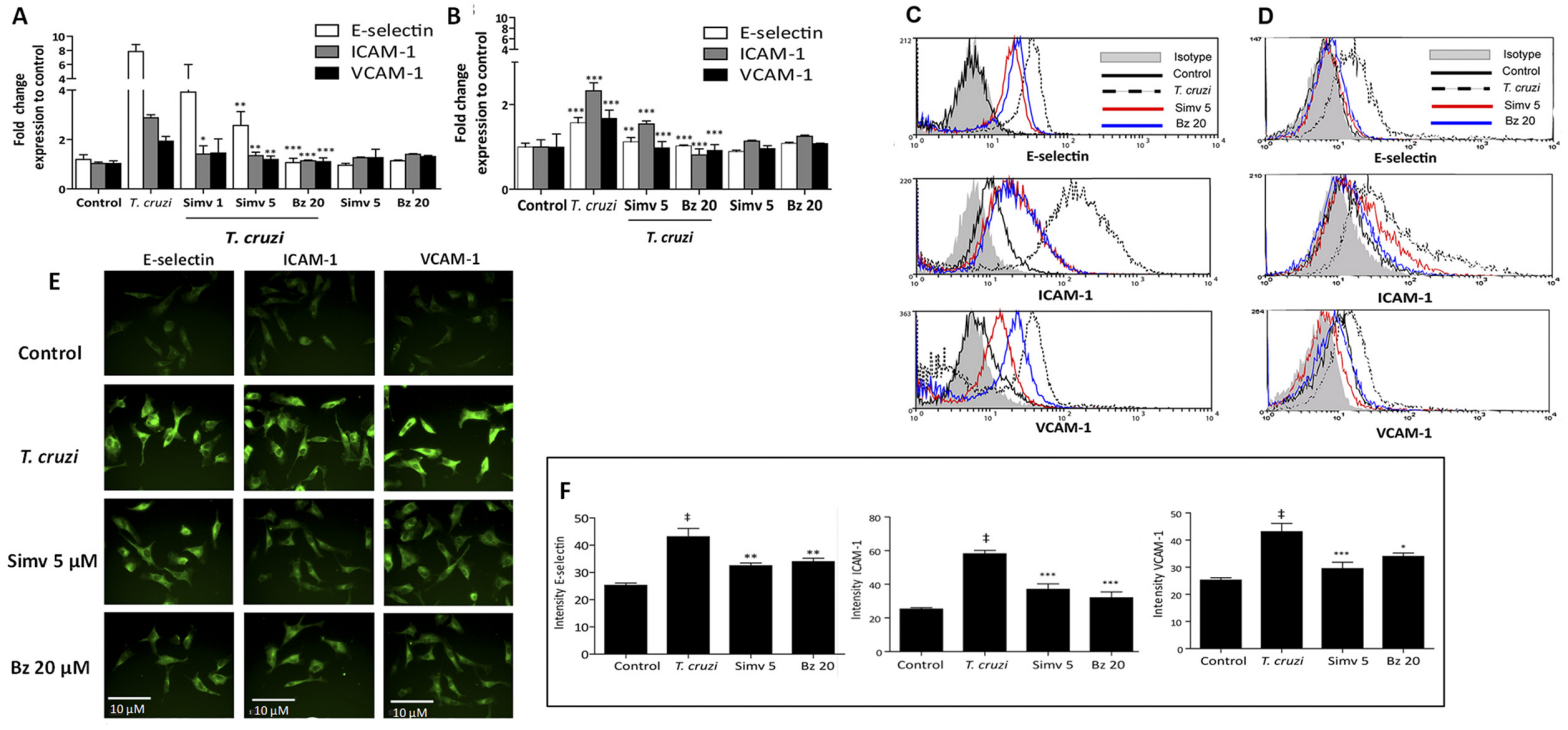
Simvastatin and benznidazole prevent cell adhesion molecule expression on endothelial cells during *Trypanosoma cruzi* infection. Endothelial-like (EA.hy926) and HUVEC cells were incubated with 5 μM simvastatin or 20 μM benznidazole dissolved in DMSO 0.025% v/v final concentration. After 24 hours, the medium was replaced, and the cells were infected for 16 hours with a 1:10 ratio of *T*. *cruzi* trypomastigotes. E-Selectin, ICAM-1, and VCAM-1 expression in EA.hy926 cells (A, C) and HUVECs (B, D) was determined at the end of the infection time. Flow cytometry was performed using human antibodies conjugated with PE, FITC, and APC for E-Selectin, ICAM-1, and VCAM-1, respectively. Panels A and B represent mean fluorescence intensity (MFI) calculations and panels C and D demonstrate representative histograms. Each group was compared with the respective IgG1 and IgG2A isotype match and normalized. In E and F, EA.hy926 cells were washed with PBS and incubated with IgG anti human E-selectin, ICAM-1, and VCAM-1 antibodies. Subsequently, the cells were incubated with anti-IgG mouse FITC-conjugated secondary antibodies. E. Representative immunofluorescence images (40X) and F. Quantitative analysis of relative fluorescence for each adhesion molecule. All controls were incubated with DMSO vehicle alone. The data are expressed as the mean ± SD from three independent experiments. The data represent the mean ± SD of MFI values (A and B) from four independent experiments. Comparisons were made by two-way ANOVA and Tukey’s post-test analysis. *** *p*<0.001; ** *p*<0.01; ‡ *p*< 0.001 (F).

**Fig 3:**
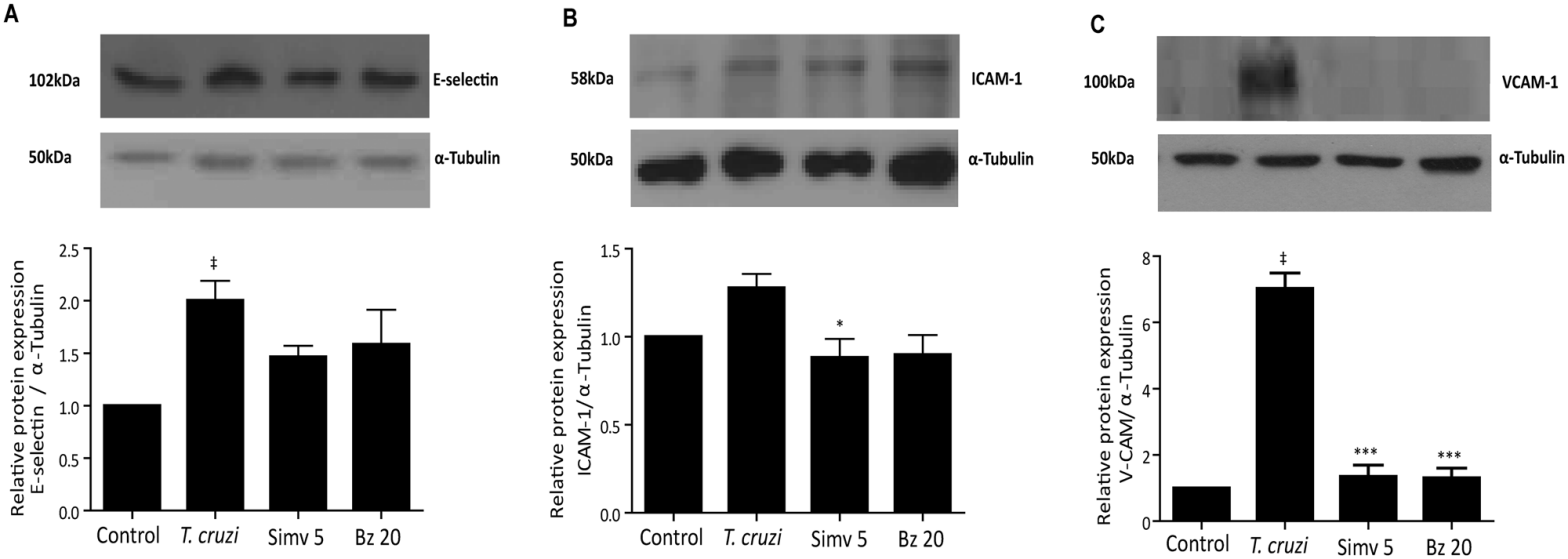
Simvastatin and benznidazole decreased cell adhesion molecule amount during *Trypanosoma cruzi* infection of endothelial cells. Endothelial-like (EA.hy926) and HUVEC cells were incubated with 5 μM simvastatin or 20 μM benznidazole 0.025% v/v final concentration. After 24 hours, the medium was replaced, and the cells were infected at a MOI of 10. After 16 hours of infection, cells were harvested, and total protein expression was determined by Western blot. The data are presented as total of E-Selectin (**A**), VCAM-1 (**B**) and ICAM-1 (**C**) content. Upper panels correspond to representative blot images. Bottom panels correspond to relative blot quantification using β-tubulin as a control. Controls were incubated with DMSO vehicle alone. The data are expressed as the mean ± SD from three independent experiments. One-way ANOVA and Tukey’s post-test analysis were used to assess significant differences. ‡ *p*<0.001 vs. control; * *p*<0.05; *** *p*< 0.001 vs. *T*. *cruzi*.

To evaluate the effect of simvastatin or benznidazole on ECAMs expression EA.hy926 and HUVEC cells were incubated with 5 μM simvastatin or 20 μM benznidazole for 24 hours and then were infected with *T*. *cruzi* trypomastigotes for 16 hours (Fig 2). The simvastatin and benznidazole concentrations used here provided the best effect on ECAM expression without cytotoxic effects on endothelial cells, either cytoskeletal alterations or on cell viability (Table 1, S2 and S3 Figs) [22]). Nevertheless, even at concentrations as low as 1 μM, the decrease in ECAM expression was observed (Fig 2A). Both drugs significantly prevented E-selectin, ICAM-1, and VCAM-1 expression as evidenced by flow cytometry (Fig 2A–2D) and immunofluorescence (Figs 2E and 3F) (Two-way ANOVA with Tukey post-test, p >0.01).

**Table 1:** Effect of simvastatin or benznidazol on cellular viability*.

	EA.hy926 cells	HUVEC	Trypomastigotes Dm28c	HL-60 cells
Simvastatin	65.36 ± 1.02	84.42 ± 1.56	4.2 ± 1.06	67.95 ± 1.11
Benznidazole	<100	<100	20.8 ± 1.35	<100

*Data expressed as the mean of IC_50_ (μM) ± SD, n = 3 independent MTT assays.

We expected that the effect of simvastatin on ECAMs would complement the trypanocidal action of benznidazole. Thus, studying their combination was necessary. To study the effect of the combination of simvastatin and benznidazole on E-selectin, ICAM-1, and VCAM-1 expression, EA.hy926 and HUVEC cells were incubated with varying concentrations of both drugs, using the same procedure as for Fig 2. For all three ECAM evaluated, at any combinatory point the CI values were >5. Thus, accordingly to Chou and Talalay these values are indicative of antagonism [20].

**Fig 4:**
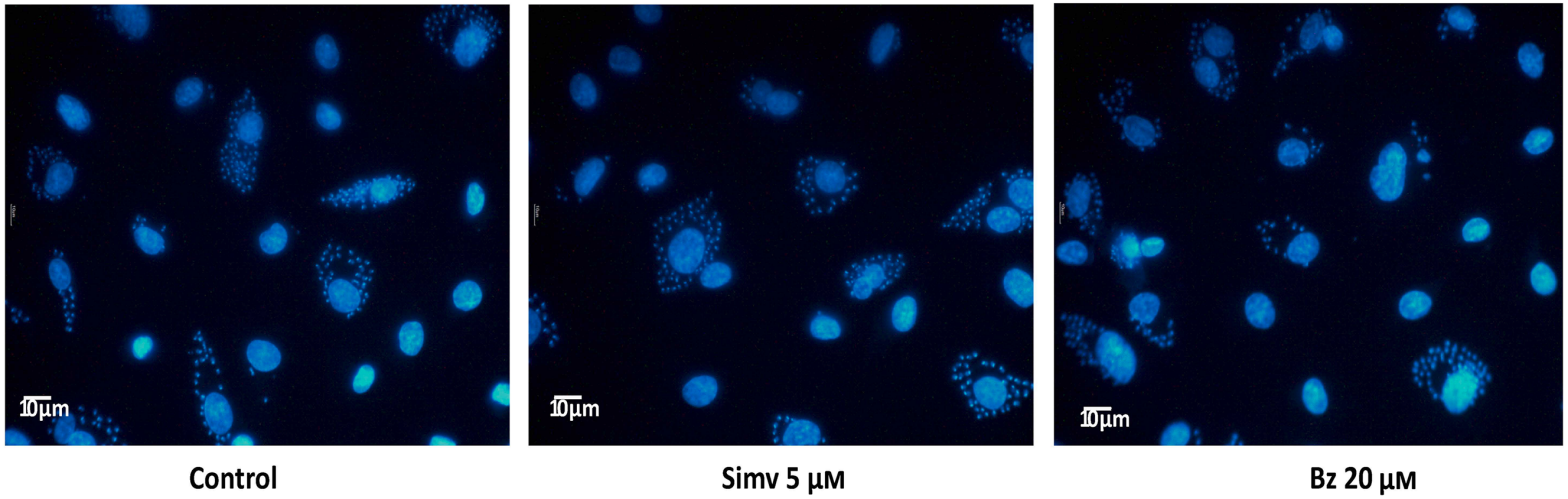
Intracellular *T*. *cruzi* load is not affected by simvastatin or benznidazole treatment before cell infection. Endothelial-like (EA.hy926) and HUVEC cells were incubated with 5 μM simvastatin or 20 μM benznidazole 0.025% v/v final concentration. After 24 hours, the medium was replaced, and the cells were infected at a MOI of 10. After 16 hours of infection, cells were washed twice with fresh medium and the drug-free medium was changed daily. Then, after 72 hours of culture, the cells were fixed in cold methanol, and nuclei were stained with DAPI. Each panel is a representative micrograph (40X) of *T*. *cruzi*-infected, DAPI stained cells. Each experimental condition was performed in duplicate and, for each duplicate, 10 photographs were taken. In average, there were counted 20.12±3.8 cells per photograph. Infected cells averaged 5.57±1.16 and healthy cells were 14.92±3.1. Infected to uninfected cells ration was 1:5. Controls were incubated with DMSO vehicle alone. The data are expressed as the mean ± SD from three independent experiments. NS: not significant after one-way ANOVA analysis.

A decrease in endothelial adhesion molecule expression, as a result of simvastatin or benznidazole administration, might be explained by intracellular sequestration of these molecules. If this is the case, total protein levels would be similar to those of uninfected cells. In Fig 3, total E-selectin (Fig 3A), ICAM-1 (Fig 3B) and VCAM-1 (Fig 3C) protein expression analyses are shown. When compared with infected, untreated cells, a slight decrease in total E-selectin protein expression was observed. This effect was more evident and significant with VCAM-1 and ICAM-1 (One-way ANOVA with Tukey post-test, p<0.05), where total protein content returned to values similar to uninfected controls. Thus, simvastatin or benznidazole administration prevented *T*. *cruzi*-triggered activation of intracellular mechanisms, which explains the increased ECAM expression on the surface of EA.hy926 cells. Conversely, the lack of response in adhesion molecule expression could obey eventually to a trypanocidal effect of simvastatin or benznidazole. Therefore, the burden of intracellular amastigotes in simvastatin or benznidazole-treated and subsequently *T*. *cruzi*-infected EA.hy926 cells was determined 72 hours after establishing the infection. To evaluate parasite load, DAPI-stained cells were photographed (20.12 ± 3.8 cells per photograph, 20 photographs per experimental condition; infected to uninfected cells ratio of 1:5) and the number of intracellular amastigotes was determined by manual count. As demonstrated in Fig 4 and S4 Fig, intracellular amastigote content is not influenced by drug pretreatment.

**Fig 5:**
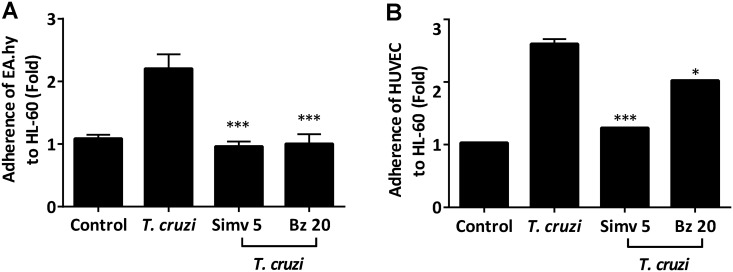
Cell adhesion was decreased by simvastatin and benznidazole during *Trypanosoma cruzi* infection. EA.hy926 cells (A) or HUVECs (B) were incubated with 5 μM simvastatin and 20 μM benznidazole 0.025% v/v final concentration. After 24 hours, the medium was replaced, and the cells were infected at a MOI of 10. After 16 hours of infection, HL-60 leukocytes that were loaded with Leuko Tracker were added to the EA.hy926 monolayer and incubated for 1.5 hours. After washing with PBS, cells were lysed, and fluorescence was measured at 480/520 nm. Controls were incubated with DMSO vehicle alone. Graphs represent the mean ± SD fold change of the relative fluorescence from three independent experiments. One-way ANOVA and Tukey’s post-test analysis were used to assess significant differences. * *p*<0.05; *** *p*<0.001.

Administration of simvastatin and benznidazole prior to *T*. *cruzi* infection prevented endothelial activation and the subsequent increase in adhesion molecule expression. Hence, it is important to clarify whether these drugs affect cell adherence. Consequently, an adhesion assay was performed using HL-60 leukocytes loaded with Leuko Tracker, which were co-incubated with *T*. *cruzi*-infected endothelial cells. The results of the analysis are demonstrated in Fig 5. Indeed, when leukocyte adhesiveness was assessed, there was a significant increase in cell adhesion to the *T*. *cruzi*-infected endothelial cells (One-way ANOVA and Tukey post-test, p<0.05). However, upon simvastatin or benznidazole treatment the adhesiveness decreased and reached similar values compared to those observed in uninfected cells. This finding is important because it links the decreased adhesion molecule expression with the physiological consequence of efficiently reducing leukocyte adhesion.

### Simvastatin and benznidazole block NF-κB activation in *Trypanosoma cruzi*-infected EA.hy926 endothelial-like cells

**Fig 6:**
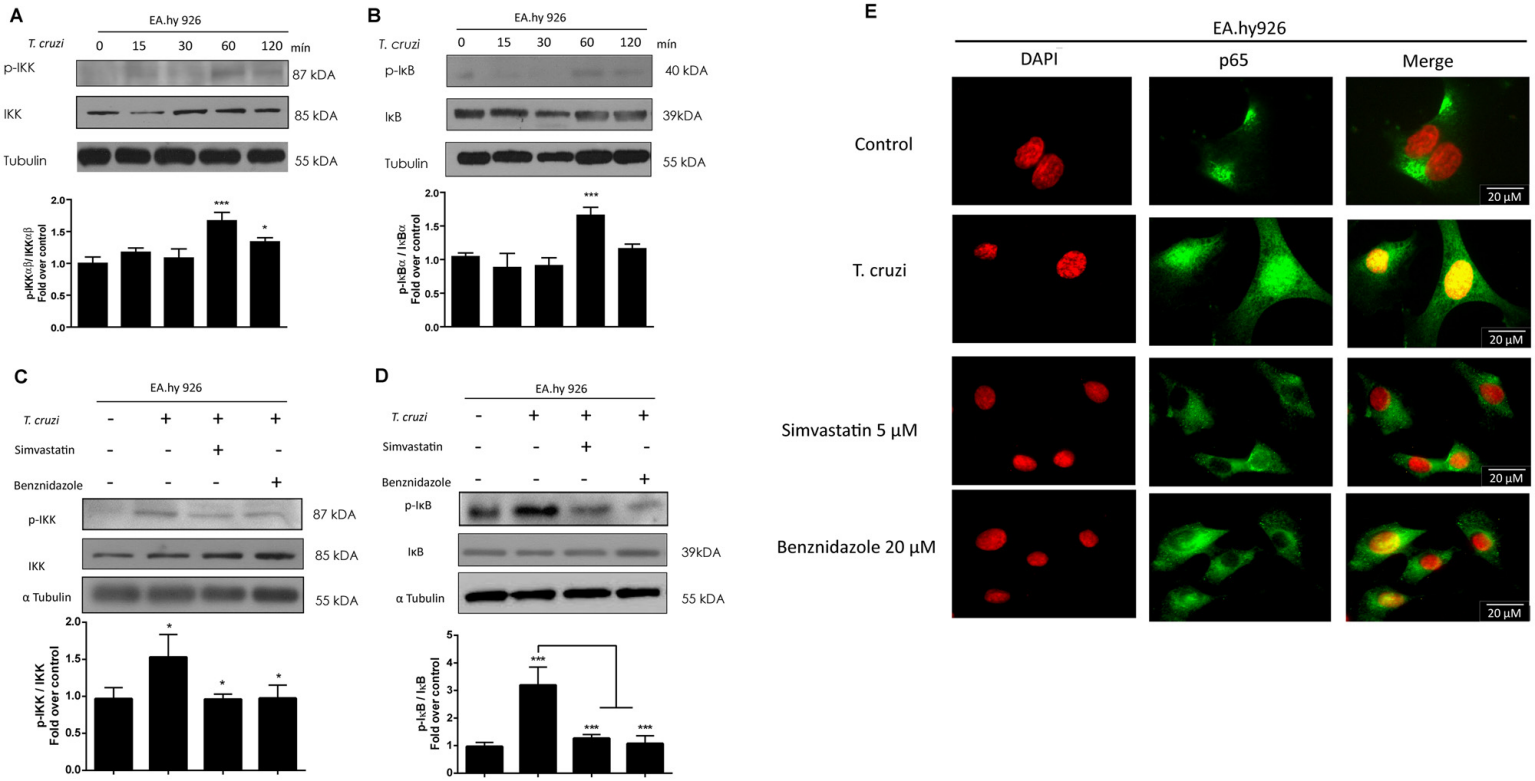
Simvastatin and benznidazole block NF-κB activation in *Trypanosoma cruzi* infection of EA.hy926 endothelial-like cells. Endothelial-like (EA.hy926) and HUVEC cells were incubated with 5 μM simvastatin or 20 μM benznidazole 0.025% v/v final concentration. After 24 hours, the medium was replaced with drug-free medium, and the cells were at a MOI of 10 for 1 hour. Then, the cells were harvested at the time points indicated in the figure, and total protein expression was determined by Western blot using rabbit monoclonal antibodies against human IKK, p-IKK, IκB, and p-IκB. Representative blots are shown for p-IKK and IKK in untreated (A) and treated (C) cells and for p-IkB and IkB in untreated (B) and treated (D) cells. Bottom panels in A-D correspond to relative blot quantification using α-tubulin as a control. All controls were incubated with DMSO vehicle alone. The data are expressed as the mean ± SD from three independent experiments. One-way ANOVA and Tukey’s post-test analysis were used to find significant differences. ‡ *p*<0.001 vs. control; * *p*<0.05; *** *p*< 0.001 vs. *T*. *cruzi*. E. Representative photographs of the p65 localization by confocal microscopy after contact of EA.hy926 cells with *T*. *cruzi* for 1 hour with or without previous 5 μM simvastatin and 20 μM benznidazole treatment.

NF-κB pathway is involved is several inflammatory events, including sepsis, where endothelial activation is induced [23]. In addition, statins decrease endothelial inflammation as part of their pleiotropic effects. Thus, simvastatin could decrease ECAM expression by affecting NF-κB pathway. To evaluate the effect of simvastatin and benznidazole upon NF-κB pathway activation, we assessed total and phosphorylated IKK and IkB protein levels by Western blot analysis (Fig 6). After 60 minutes of incubating endothelial cells with *T*. *cruzi* trypomastigotes, the respective phosphorylated IKK form increased significantly compared with non-stimulated cells (Fig 6A) (One-way ANOVA and Tukey post-test, p<0.05). Similarly, p-IkB increased at the same time point (Fig 6B). When the endothelial cells were incubated with simvastatin or benznidazole after previous *T*. *cruzi* incubation, these two drugs significantly decreased the response of the Ikk-IkB system (Fig 6C and 6D) (One-way ANOVA and Tukey post-test, p<0.05). Thus, NF-κB is modulated by simvastatin and benznidazole, which prevents the activation of these two essential NF-κB pathway proteins during a challenge with the parasite. This is demonstrated by decreased nuclear p65 localization when infected endothelial cells are previously incubated with simvastatin or benznidazole (Fig 6E). Aside from nuclear p65 localization after *T*. *cruzi* challenge, cytosolic p65 levels were also increased compared with uninfected controls.

### 15-epi-lipoxin A4 mediated the effects of simvastatin

**Fig 7:**
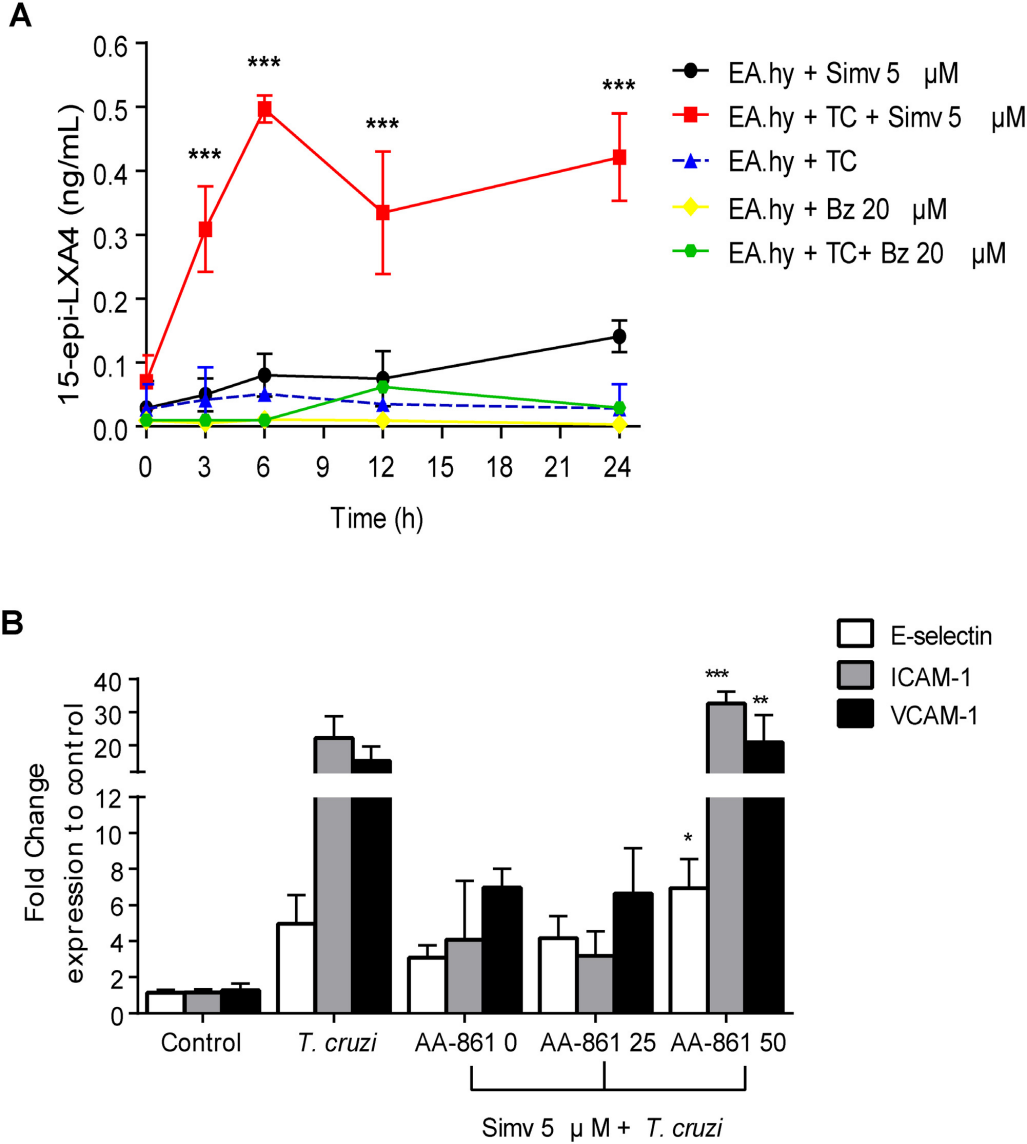
Simvastatin-induced 15-epi-lipoxin A4 mediates cell adhesion molecule expression after endothelial activation by *Trypanosoma cruzi* infection. A) EA.hy926 cells were infected with *T*. *cruzi* trypomastigotes (Dm28c) at a MOI of 10 for 16 hours. After washing with fresh culture medium, 5 μM simvastatin was added to the cells and harvested at the time point indicated. 15-epi-lipoxin A4 content was determined in the supernatants by competitive ELISA. The data are expressed as the mean ± SD from three independent experiments. Statistical analysis was performed using ANOVA compared with zero time. * *P*<0.05; ***p<0.0001. B) EA.hy926 cells were incubated with 5 μM simvastatin and 25 and 50 μM AA-861 (a competitive 5-lipoxygenase inhibitor) for 24 hours. After washing the cells, they were infected with *T*. *cruzi* trypomastigotes (Dm28c) at a 1:10 ratio for 16 hours. ECAM expression was determined on the EA.hy926 cell surface by flow cytometry using human antibodies conjugated with PE, FITC, and APC for E-Selectin, ICAM-1, and VCAM-1, respectively. Controls were incubated with DMSO vehicle alone. Results represent the normalized mean fluorescent intensity (MFI) ± SD from three independent experiments. One-way ANOVA and Tukey’s post-test analysis were used to assess significant differences *p<0.01; *** *p*<0.001 vs. *T*. *cruzi*.

Among the pleiotropic effects of statins, their anti-inflammatory actions include the induction of pro-resolutive, anti-inflammatory molecules such as 15-epi-LXA4, which may mediate the decreased leukocyte adhesion [24]. Indeed, simvastatin induced 15-epi-LXA4 in *T*. *cruzi*-infected endothelial cells. In Fig 7A and Table 2, 5 μM simvastatin was added after incubation of the EA-hy926 cells with *T*. *cruzi* trypomastigotes for 16 hours. 15-epi-LXA4 levels rose slightly with simvastatin alone (Fig 7A); while the *T*. *cruzi* infection progressed, there was a significant increase in 15-epi-LXA4 levels (Fig 7A and Table 1) (One-way ANOVA and Tukey post-test, p<0.001). In this experiment, the model that had been used so far was modified by adding drug treatment after establishment of the infection. Notwithstanding, the most important fact is that the simvastatin did not induce the production of this eicosanoid in the absence of an activating factor of endothelial cells such as the parasite. In contrast, when 5-lipoxygenase activity is inhibited in the presence of a competitive inhibitor (AA-861) [25], ICAM expression was restored (Fig 7B). Although AA-861 concentration appears high (50 μM), there are several reports that used this inhibitor at concentrations as high as 100 μM, without reporting off-target effects [26–29]. Thus, there is a connection between the increased 15-epi-LXA4 and the action of 5-lipooxygenase when simvastatin was administered.

**Table 2:** Mass spectra data and quantification of 15-epi-lipoxin A4 determined by DSA-TOF.

Experimental condition		[M-H]^-^	Calculated m/z	Error (ppm)	Formula	Concentration (ng/mL)
						Mean ± SD
EA.hy926 cells		351.2171	351.2170	0.3	C20H32O5	0.14 ± 0.02
*T*. *cruzi*		351.2171	351.2190	-5.4	C20H32O5	0.32 ± 0.07
EA.hy926 + *T*. *cruzi*		351.2171	351.2183	-3.4	C20H32O5	0.5 ± 0.14
EA.hy926 + *T*. *cruzi*	Simv 3 hours	351.2171	351.2126	12.8	C20H32O5	1.58 ± 0.21
EA.hy926 + *T*. *cruzi*	Simv 6 hours	351.2171	351.2180	-2.6	C20H32O5	3.58 ± 1.22
EA.hy926 + *T*. *cruzi*	Simv 12 hours	351.2171	351.2161	2.8	C20H32O5	37.28 ±11.23 ***
EA.hy926 + *T*. *cruzi*	Simv 24 hours	351.2171	351.2169	0.6	C20H32O5	326 ± 34.25 ***

*** p value < 0.0001

**Fig 8:**
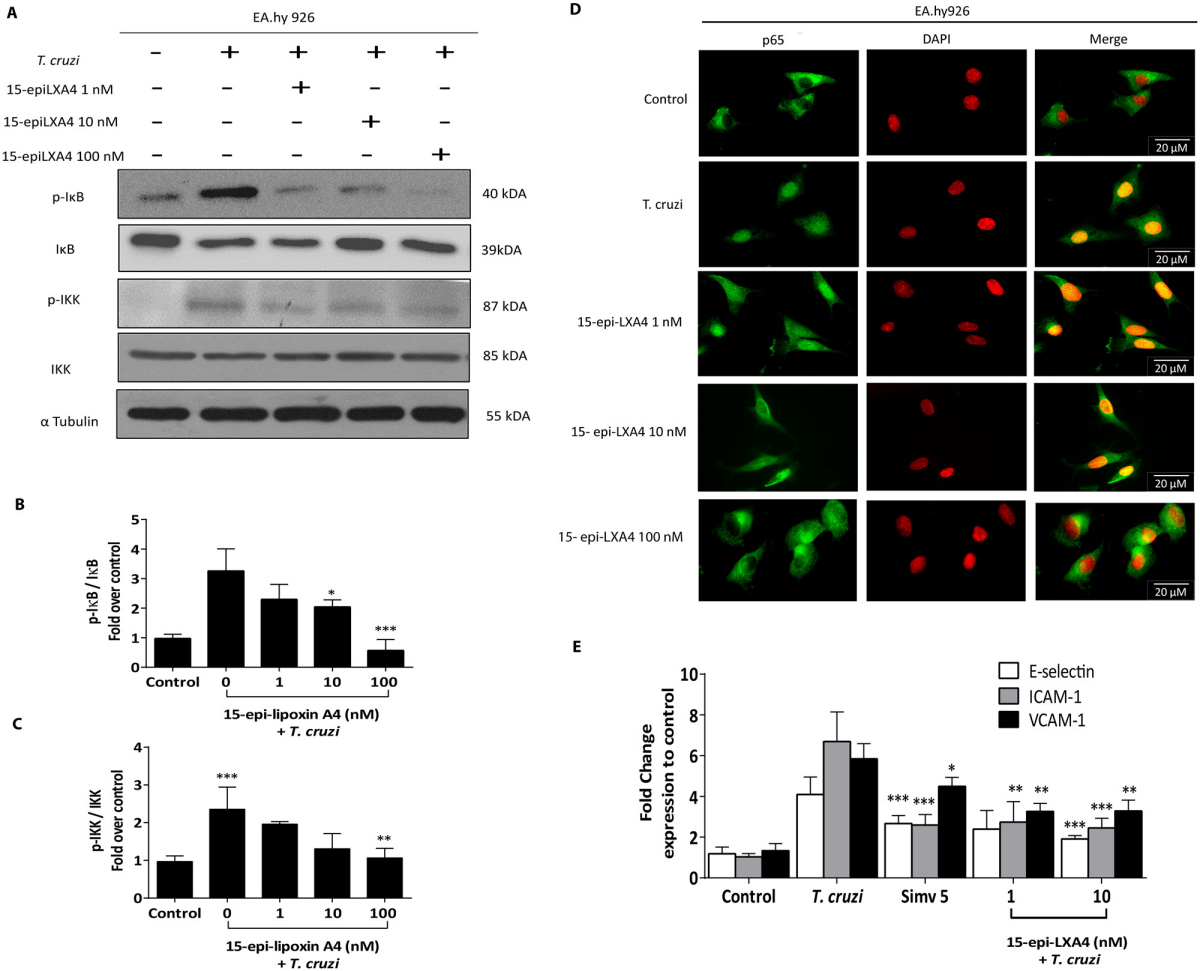
15-epi-lipoxinA4 prevents ECAMs expression on EA.hy926 during *Trypanosoma cruzi* infection and blocks NF-κB activation. A. EA.hy926 cells were incubated with 15-epi-LXA4 at the concentrations indicated in the figure. After 24 hours, the medium was replaced, and the cells were infected with a 1:10 ratio of *T*. *cruzi* trypomastigotes. After 1 hour, the cells were harvested, and total protein expression was determined by Western blot using rabbit monoclonal antibodies against human IKK, p-IKK, IκB, and p-IκB. B-C correspond to the relative blot quantification using α-tubulin as a control. The data are expressed as the mean ± SD from three independent experiments. One-way ANOVA and Tukey’s post-test analysis were used to find significant differences. ‡ *p*<0.001 vs. control; * *p*<0.05; *** *p*<0.001 vs. *T*. *cruzi*. D. Representative photographs of p65 localization by confocal microscopy after EA.hy926 cell contact with *T*. *cruzi* for 1 hour with or without previous 15-epi-LXA4 treatment. E. EA.hy926 cells were incubated with 1 and 10 nM 15-epi-LXA4. After 24 hours, cells were infected with *T*. *cruzi* trypomastigotes at a 1:10 ratio for 16 hours. ECAM expression was determined on the EA.hy926 cell surface by flow cytometry using human antibodies conjugated with PE, FITC, and APC for E-Selectin, ICAM-1, and VCAM-1, respectively. All controls were incubated with DMSO vehicle alone. Results represent the normalized mean fluorescent intensity (MFI) ± SD from three independent experiments. One way ANOVA and Tukey’s post-test analysis were used to assess significant differences *p<0.05; **p<0.01; ***p<0.001 vs. *T*. *cruzi*.

The addition of exogenous 15-epi-LXA4 before endothelial cell infection, as has been done in the other experiments with simvastatin, decreased IKK-IκB pathway activity in a dose-dependent fashion (Fig 8A–8C). Furthermore, 100 nM 15-epi-LXA4 at the highest concentration used decreased nuclear p65 migration (Fig 8D). Thus, NF-κB pathway activity is decreased in a similar manner as simvastatin. In fact, 15-epi-LXA4 similarly reduced endothelial adhesion molecule expression (Fig 8E).

## Discussion

In Chagas cardiomyopathy, there is an inflammatory vasculopathy, which is demonstrated by sustained expression of the adhesion molecules E-selectin, VCAM-1 and ICAM-1 during *T*. *cruzi* infection. ICAM-1 is expressed at a low level constitutively on the cell surface. ECAM expression increased significantly only after inflammatory stimuli such as LPS, TNF-α [30,31] or intracellular infection with *T*. *cruzi* [32], which tends to be sustained over time.

Furthermore, according to the results reported here, both simvastatin and benznidazole prevented the increased expression of these molecules as *T*. *cruzi* infection was installed. Indeed, decreased expression of CAMs had functional consequences. Leukocyte adhesion was also decreased by both simvastatin and benznidazole. Albeit this functional decrease in cell adhesion is more evident with simvastatin, the most striking result is the action of benznidazole. The reduction in CAM expression and, consequently, cell adhesion was independent of the trypanocidal activity of benznidazole. Indeed, the anti-inflammatory effect of benznidazole had already been reported [33]. In experimental sepsis models, benznidazole attenuated NF-κB and the MAPK pathway activities, highlighting its immunomodulatory capacity [34,35]. Undoubtedly, these models did not attend the antiparasitic capacity of benznidazole. Neither did our experiments. Herein, we report its ability to prevent endothelial activation during *T*. *cruzi* infection for the first time. That is a critical factor to support the usefulness of this drug for treatment during the chronic phase of CD.

In our experimental model, simvastatin was administered before producing infection with *T*. *cruzi*. Then, the culture medium was changed to fresh medium without drug and incubated the endothelial cells with the parasite for 16 hours. Therefore, it is unlikely that the drug presents trypanocidal activity because it was not present when the infection occurred. The idea of this model is to cause an environment where the infection cannot be spread, due to the action of simvastatin on endothelium activation. However, simvastatin and other 3-hydroxy-3-methylglutaryl-coenzyme A (HMGCoA reductase) inhibitors can inhibit parasite growth, especially in the replicative forms. In the parasite, this enzyme is essential for mevalonate and ergosterol synthesis, and is inhibited by statins, affecting parasite viability. In the work reported by Silva et al. [36], simvastatin decreased epimastigote proliferation but at concentrations in the millimolar range. Apart from this observation, our results support the findings of the mentioned report since the reduction of endothelial activation is only part of the broad anti-inflammatory effect of simvastatin in that murine model of acute CD.

Previously, we reported that benznidazole prevented endothelial damage in a murine model of chronic Chagas heart disease [6]. However, the effect of simvastatin on cellular models of *T*. *cruzi*-induced endothelial activation was not yet studied. It was interesting to find that cytosolic p65 levels were increased after *T*. *cruzi* challenge. This finding suggests that this protein could be upregulated during an inflammatory drive. However, the most outstanding result is that both simvastatin and benznidazole decreased NF-κB pathway activation through IKK-IκB pathway inactivation and decreasing nuclear migration of p65. Nonetheless, a small fraction of p65 remained in the nuclei of benznidazole treated cells, and to a lesser extent, in cells that had been incubated with simvastatin. Thus, despite drug treatment, an inflammatory drive might persist due to this low-grade NF-κB activation. These findings are supported by previous reports in other experimental models [34,35,37]. Furthermore, according to our results, the effect of simvastatin on this signal transduction pathway and finally on adhesion molecule expression took place through 15-epi-lipoxin A4 generation. This eicosanoid decreased p65 migration towards endothelial cell nuclei similar to simvastatin. However, benznidazole did not induce 15-epi-lipoxin A4 production (Fig 7A); therefore, the mechanism of IKK-IκB pathway inhibition could be addressed through another yet unknown mechanism.

Considering that simvastatin and benzidazole share similar effects on ECAM expression and NF-κB pathway, it is possible to think that their combined effect could be synergistic. Unexpectedly, the effect was rather antagonistic. The explanations for antagonism are diverse. It is possible that two drugs acting on the same target may behave as antagonistic [38,39]. Thus, our findings should not be surprising since both drugs interfere in the same signaling pathway, NFκB pathway. In any case, it is necessary to consider that both drugs act within a complex network of biological functions. Thus, it is not easy to conclude, based on an *in vitro* model, if this antagonism is important in a living model of CD. In any case, this interaction should not invalidate our findings for benznidazole. In this report, we confirm the involvement of benznidazole in the NFkB pathway, and through this pathway, its modulation of the expression of ECAMs in endothelial cells infected with *T*. *cruzi*, regardless of its trypanocidal capacity.

The role of pro-resolving lipids in *T*. *cruzi*-induced inflammatory processes is becoming increasingly important to understand chagasic cardiomyopathy [40,41]. The role of LTB_4_ and PAF as produced by macrophages has already been reported to control parasitemia in *in vivo* CD models [40,42]. LTB4, which is dependent on 5-LO activity, is involved in the decrease in inflammation, collagen deposition and lymphocyte migration to the myocardium [40]. In addition, 5-LO derivatives are increasingly associated with acute inflammatory process resolution [43–46]. Thus, these mediators could be more involved in the acute phase of Chagas disease. However, in a chronic model of Chagas cardiomyopathy, simvastatin decreased inflammation [11]. Most likely, simvastatin, by inducing 15-epi-LXA4, prevented leukocyte migration into the myocardium by decreasing endothelial activation [44], thus contributing to reduced myocardial damage. In any case, it is important to verify this hypothesis in future studies using *in vivo* CCC models. It is also important to consider the immunomodulatory and anti-inflammatory role of benznidazole in Chagas cardiomyopathy progression.

In conclusion, simvastatin and benznidazole prevent endothelial activation, as demonstrated by decreased expression of the adhesion molecules E-selectin, ICAM-1, and VCAM-1, which involves decreasing NF-κB pathway activity, and at least for the case of simvastatin, by increased 15-epi-LXA4 production.

## Supporting Information

S1 Fig Representative DSA—TOF mass spectra for standard 15-epi-LXA4. 15-epi-LXA4 was serially diluted in PBS and measured by DSA-TOF to generate a standard curve to quantify 15-epi-LXA4 concentrations in experimental samples. Compound identification was done using DSA/TOF and AxION software. The inset picture shows the standard curve used to calculate the 15-epi-LXA4 concentrations in the supernatants of the experimental samples. Points represent the mean, and the error bars represent the standard deviation of three replicates.
(TIF)

S2 Fig Effect of simvastatin on cytoskeleton. Cells were treated with vehicule (0.1% v/v DMSO) or simvastatin (1, 5, 7.5 and 10 μM) during 24 hours. A. Cells were fixed, permeabilized, stained with rhodamine-phalloidin and visualized by fluorescence microscopy (40X). B. Graphics analyses disrupted cytoskeleton cell percent. Data are shown as mean ± SD (n = 3 independent experiments). *** p<0,001 vs control.
(TIF)

S3 Fig Effect of simvastatin on cell viability evaluated by flow cytometry. Cells were treated with vehicule control (0.1% v/v DMSO) or simvastatin (1, 5, 7.5 and 10 μM) during 24 hours. After 24hof incubation cells were stained with propidium iodide (0.5 μM) A. Representative histograms are shown for simvastatin-treated and control cells. Results are shown as the percentage of cells PI+. B. Graphs shown the histograms analyses.
(TIF)

S4 Fig Intracellular *T*. *cruzi* load is not affected by simvastatin or benznidazole treatment before cell infection—Quantitative analysis. Endothelial-like (EA.hy926) and HUVEC cells were incubated with 5 μM simvastatin or 20 μM benznidazole 0.025% v/v final concentration. After 24 hours, the medium was replaced, and the cells were infected at a MOI of 10. After 16 hours of infection, cells were washed twice with fresh medium and the drug-free medium was changed daily. Then, after 72 hours of culture, the cells were fixed in cold methanol, and nuclei were stained with DAPI. The figure correspond to the quantitative comparison of the amastigote load for each experimental group. Each experimental condition was performed in duplicate and, for each duplicate, 10 photographs were taken. In average, there were counted 20.12±3.8 cells per photograph. Infected cells averaged 5.57±1.16 and healthy cells were 14.92±3.1. Infected to uninfected cells ration was 1:5. Controls were incubated with DMSO vehicle alone. The data are expressed as the mean ± SD from three independent experiments. NS: not significant after one-way ANOVA analysis.
(TIF)

## References

[1] WHO Working to overcome the global impact of neglected tropical diseases: first WHO report on neglected tropical diseases: update 2011. Geneva: World Health Organization 2011 viii, 14 p. p.

[2] Coura JR. Chagas disease: control, elimination and eradication. Is it possible? Mem Inst Oswaldo Cruz 2013; 108: 962–967. 10.1590/0074-0276130565 24402148 PMC4005548

[3] Rassi A Jr., Rassi A, Marcondes de Rezende J. American trypanosomiasis (Chagas disease). Infect Dis Clin North Am 2012; 26: 275–291. 10.1016/j.idc.2012.03.002 22632639

[4] Ribeiro AL, Nunes MP, Teixeira MM, Rocha MO. Diagnosis and management of Chagas disease and cardiomyopathy. Nat Rev Cardiol 2012; 9: 576–589. 10.1038/nrcardio.2012.109 22847166

[5] Andrade D, Serra R, Svensjo E, Lima AP, Ramos ES Jr., et al *Trypanosoma cruzi* invades host cells through the activation of endothelin and bradykinin receptors: a converging pathway leading to Chagasic vasculopathy. Br J Pharmacol 2012; 165: 1333–1347. 10.1111/j.1476-5381.2011.01609.x 21797847 PMC3372720

[6] Molina-Berrios A, Campos-Estrada C, Lapier M, Duaso J, Kemmerling U, et al Benznidazole prevents endothelial damage in an experimental model of Chagas disease. Acta Trop 2013; 127: 6–13. 10.1016/j.actatropica.2013.03.006 23529066

[7] Huang H, Petkova SB, Cohen AW, Bouzahzah B, Chan J, et al Activation of transcription factors AP-1 and NF-kappa B in murine Chagasic myocarditis. Infect Immun 2003; 71: 2859–2867. 12704159 10.1128/IAI.71.5.2859-2867.2003 PMC153290

[8] Prado CM, Jelicks LA, Weiss LM, Factor SM, Tanowitz HB, et al The vasculature in Chagas disease. Adv Parasitol 2011; 76: 83–99. 10.1016/B978-0-12-385895-5.00004-9 21884888 PMC3557505

[9] Rossi MA, Tanowitz HB, Malvestio LM, Celes MR, Campos EC, et al Coronary microvascular disease in chronic Chagas cardiomyopathy including an overview on history, pathology, and other proposed pathogenic mechanisms. PLoS Negl Trop Dis 2010; 4: e674 10.1371/journal.pntd.0000674 20824217 PMC2930857

[10] Molina-Berrios A, Campos-Estrada C, Henriquez N, Faundez M, Torres G, et al Protective role of acetylsalicylic acid in experimental *Trypanosoma cruzi* infection: evidence of a 15-epi-lipoxin A(4)-mediated effect. PLoS Negl Trop Dis 2013; 7: e2173 10.1371/journal.pntd.0002173 23638194 PMC3630130

[11] Melo L, Caldas IS, Azevedo MA, Goncalves KR, da Silva do Nascimento AF, et al Low doses of simvastatin therapy ameliorate cardiac inflammatory remodeling in *Trypanosoma cruzi*-infected dogs. Am J Trop Med Hyg 2011; 84: 325–331. 10.4269/ajtmh.2011.10-0451 21292909 PMC3029192

[12] Birnbaum Y, Ye Y, Lin Y, Freeberg SY, Huang MH, et al Aspirin augments 15-epi-lipoxin A4 production by lipopolysaccharide, but blocks the pioglitazone and atorvastatin induction of 15-epi-lipoxin A4 in the rat heart. Prostaglandins Other Lipid Mediat 2007; 83: 89–98. 17259075 10.1016/j.prostaglandins.2006.10.003

[13] Aranda E, Owen GI. A semi-quantitative assay to screen for angiogenic compounds and compounds with angiogenic potential using the EA.hy926 endothelial cell line. Biol Res 2009; 42: 377–389. doi: /S0716-97602009000300012 19915746

[14] Collins SJ, Gallo RC, Gallagher RE. Continuous growth and differentiation of human myeloid leukaemic cells in suspension culture. Nature 1977; 270: 347–349. 271272 10.1038/270347a0

[15] Contreras VT, Araujo-Jorge TC, Bonaldo MC, Thomaz N, Barbosa HS, et al Biological aspects of the Dm 28c clone of *Trypanosoma cruzi* after metacyclogenesis in chemically defined media. Mem Inst Oswaldo Cruz 1988; 83: 123–133. 3074237 10.1590/s0074-02761988000100016

[16] Stach K, Nguyen XD, Lang S, Elmas E, Weiss C, et al Simvastatin and atorvastatin attenuate VCAM-1 and uPAR expression on human endothelial cells and platelet surface expression of CD40 ligand. Cardiol J 2012; 19: 20–28. 22298164 10.5603/cj.2012.0005

[17] Yang JC, Huang F, Wu CJ, Chen YC, Lu TH, et al Simvastatin reduces VCAM-1 expression in human umbilical vein endothelial cells exposed to lipopolysaccharide. Inflamm Res 2012; 61: 485–491. 10.1007/s00011-012-0435-9 22245985

[18] Lopez-Munoz R, Faundez M, Klein S, Escanilla S, Torres G, et al *Trypanosoma cruzi*: In vitro effect of aspirin with nifurtimox and benznidazole. Exp Parasitol 2010; 124: 167–171. 10.1016/j.exppara.2009.09.005 19735656

[19] Mosmann T. Rapid colorimetric assay for cellular growth and survival: application to proliferation and cytotoxicity assays. J Immunol Methods 1983; 65: 55–63. 6606682 10.1016/0022-1759(83)90303-4

[20] Chou TC. Theoretical basis, experimental design, and computerized simulation of synergism and antagonism in drug combination studies. Pharmacol Rev 2006; 58: 621–681. 16968952 10.1124/pr.58.3.10

[21] Tong S, Neboori HJ, Tran ED, Schmid-Schonbein GW. Constitutive expression and enzymatic cleavage of ICAM-1 in the spontaneously hypertensive rat. J Vasc Res 2011; 48: 386–396. 10.1159/000323474 21464573 PMC3080588

[22] Copaja M, Venegas D, Aranguiz P, Canales J, Vivar R, et al Simvastatin disrupts cytoskeleton and decreases cardiac fibroblast adhesion, migration and viability. Toxicology 2012; 294: 42–49. 10.1016/j.tox.2012.01.011 22306966

[23] Liang Y, Li X, Zhang X, Li Z, Wang L, et al Elevated levels of plasma TNF-alpha are associated with microvascular endothelial dysfunction in patients with sepsis through activating the NF-kappaB and p38 mitogen-activated protein kinase in endothelial cells. Shock 2014; 41: 275–281. 10.1097/SHK.0000000000000116 24430552

[24] Birnbaum Y, Ye Y. Pleiotropic effects of statins: the role of eicosanoid production. Curr Atheroscler Rep 2012; 14: 135–139. 10.1007/s11883-012-0232-5 22286195

[25] Yoshimoto T, Yokoyama C, Ochi K, Yamamoto S, Maki Y, et al 2,3,5-Trimethyl-6-(12-hydroxy-5,10-dodecadiynyl)-1,4-benzoquinone (AA861), a selective inhibitor of the 5-lipoxygenase reaction and the biosynthesis of slow-reacting substance of anaphylaxis. Biochim Biophys Acta 1982; 713: 470–473. 6817808

[26] Lee HM, Ok SH, Sung HJ, Eun SY, Kim HJ, et al Mepivacaine-induced contraction involves phosphorylation of extracellular signal-regulated kinase through activation of the lipoxygenase pathway in isolated rat aortic smooth muscle. Can J Physiol Pharmacol 2013; 91: 285–294. 10.1139/cjpp-2012-0197 23627840

[27] Choi YS, Jeong YS, Ok SH, Shin IW, Lee SH, et al The direct effect of levobupivacaine in isolated rat aorta involves lipoxygenase pathway activation and endothelial nitric oxide release. Anesth Analg 2010; 110: 341–349. 10.1213/ANE.0b013e3181c76f52 19955508

[28] Kim BJ, Kim SY, Lee S, Jeon JH, Matsui H, et al The role of transient receptor potential channel blockers in human gastric cancer cell viability. Can J Physiol Pharmacol 2012; 90: 175–186. 10.1139/y11-114 22308955

[29] Lee S, Felts KA, Parry GC, Armacost LM, Cobb RR. Inhibition of 5-lipoxygenase blocks IL-1 beta-induced vascular adhesion molecule-1 gene expression in human endothelial cells. J Immunol 1997; 158: 3401–3407. 9120300

[30] Kempe S, Kestler H, Lasar A, Wirth T. NF-kappaB controls the global pro-inflammatory response in endothelial cells: evidence for the regulation of a pro-atherogenic program. Nucleic Acids Res 2005; 33: 5308–5319. 16177180 10.1093/nar/gki836 PMC1226313

[31] Norata GD, Tibolla G, Seccomandi PM, Poletti A, Catapano AL. Dihydrotestosterone decreases tumor necrosis factor-alpha and lipopolysaccharide-induced inflammatory response in human endothelial cells. J Clin Endocrinol Metab 2006; 91: 546–554. 16317058 10.1210/jc.2005-1664

[32] Dias WB, Fajardo FD, Graca-Souza AV, Freire-de-Lima L, Vieira F, et al Endothelial cell signalling induced by trans-sialidase from *Trypanosoma cruzi* . Cell Microbiol 2008; 10: 88–99. 17672865 10.1111/j.1462-5822.2007.01017.x

[33] Cutrullis RA, Moscatelli GF, Moroni S, Volta BJ, Cardoni RL, et al Benznidazole therapy modulates interferon-gamma and M2 muscarinic receptor autoantibody responses in *Trypanosoma cruzi*-infected children. PLoS One 2011; 6: e27133 10.1371/journal.pone.0027133 22066031 PMC3205037

[34] Manarin R, Pascutti MF, Ruffino JP, De Las Heras B, Bosca L, et al Benznidazole blocks NF-kappaB activation but not AP-1 through inhibition of IKK. Mol Immunol 2010; 47: 2485–2491. 10.1016/j.molimm.2010.06.002 20598748

[35] Ronco MT, Manarin R, Frances D, Serra E, Revelli S, et al Benznidazole treatment attenuates liver NF-kappaB activity and MAPK in a cecal ligation and puncture model of sepsis. Mol Immunol 2011; 48: 867–873. 10.1016/j.molimm.2010.12.021 21269697

[36] Silva RR, Shrestha-Bajracharya D, Almeida-Leite CM, Leite R, Bahia MT, et al Short-term therapy with simvastatin reduces inflammatory mediators and heart inflammation during the acute phase of experimental Chagas disease. Mem Inst Oswaldo Cruz 2012; 107: 513–521. 22666863 10.1590/s0074-02762012000400012

[37] Ortego M, Gomez-Hernandez A, Vidal C, Sanchez-Galan E, Blanco-Colio LM, et al HMG-CoA reductase inhibitors reduce I kappa B kinase activity induced by oxidative stress in monocytes and vascular smooth muscle cells. J Cardiovasc Pharmacol 2005; 45: 468–475. 15821443 10.1097/01.fjc.0000159042.50488.e5

[38] Berenbaum MC. What Is Synergy. Pharmacological Reviews 1989; 41: 93–141. 2692037

[39] Fitzgerald JB, Schoeberl B, Nielsen UB, Sorger PK. Systems biology and combination therapy in the quest for clinical efficacy. Nat Chem Biol 2006; 2: 458–466. 16921358 10.1038/nchembio817

[40] Pavanelli WR, Gutierrez FR, Mariano FS, Prado CM, Ferreira BR, et al 5-lipoxygenase is a key determinant of acute myocardial inflammation and mortality during *Trypanosoma cruzi* infection. Microbes Infect 2010; 12: 587–597. 10.1016/j.micinf.2010.03.016 20381637

[41] Machado FS, Dutra WO, Esper L, Gollob KJ, Teixeira MM, et al Current understanding of immunity to *Trypanosoma cruzi* infection and pathogenesis of Chagas disease. Semin Immunopathol 2012; 34: 753–770. 10.1007/s00281-012-0351-7 23076807 PMC3498515

[42] Talvani A, Teixeira MM. Inflammation and Chagas disease some mechanisms and relevance. Adv Parasitol 2011; 76: 171–194. 10.1016/B978-0-12-385895-5.00008-6 21884892

[43] Buckley CD, Gilroy DW, Serhan CN. Proresolving lipid mediators and mechanisms in the resolution of acute inflammation. Immunity 2014; 40: 315–327. 10.1016/j.immuni.2014.02.009 24656045 PMC4004957

[44] Chinthamani S, Odusanwo O, Mondal N, Nelson J, Neelamegham S, et al Lipoxin A4 inhibits immune cell binding to salivary epithelium and vascular endothelium. Am J Physiol Cell Physiol 2012; 302: C968–978. 10.1152/ajpcell.00259.2011 22205391 PMC3330736

[45] Serhan CN. Lipoxins and aspirin-triggered 15-epi-lipoxins are the first lipid mediators of endogenous anti-inflammation and resolution. Prostaglandins Leukot Essent Fatty Acids 2005; 73: 141–162. 16005201 10.1016/j.plefa.2005.05.002

[46] Machado FS, Mukherjee S, Weiss LM, Tanowitz HB, Ashton AW. Bioactive lipids in *Trypanosoma cruzi* infection. Adv Parasitol 2011; 76: 1–31. 10.1016/B978-0-12-385895-5.00001-3 21884885 PMC3564251

