# Supplementary figures and images for: Simvastatin and Benznidazole-Mediated Prevention of Trypanosoma cruzi-Induced Endothelial Activation: Role of 15-epi-lipoxin A4 in the Action of Simvastatin
Source: PLoS Negl Trop Dis. 2015 May 15;9(5):e0003770. doi: 10.1371/journal.pntd.0003770 (PMC4433340; doi:10.1371/journal.pntd.0003770)

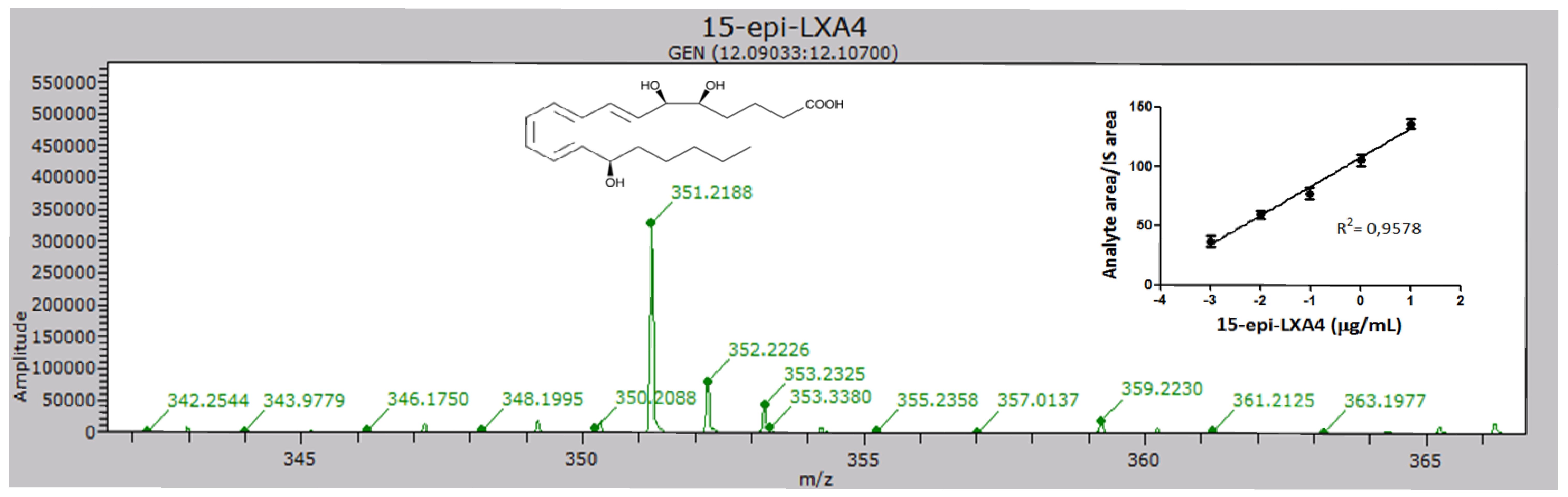

Supplement: S1 Fig — 15-epi-LXA4 was serially diluted in PBS and measured by DSA-TOF to generate a standard curve to quantify 15-epi-LXA4 concentrations in experimental samples. Compound identification was done using DSA/TOF and AxION software. The inset picture shows the standard curve used to calculate the 15-epi-LXA4 concentrations in the supernatants of the experimental samples. Points represent the mean, and the error bars represent the standard deviation of three replicates. (TIF) [file pntd.0003770.s001.tif]

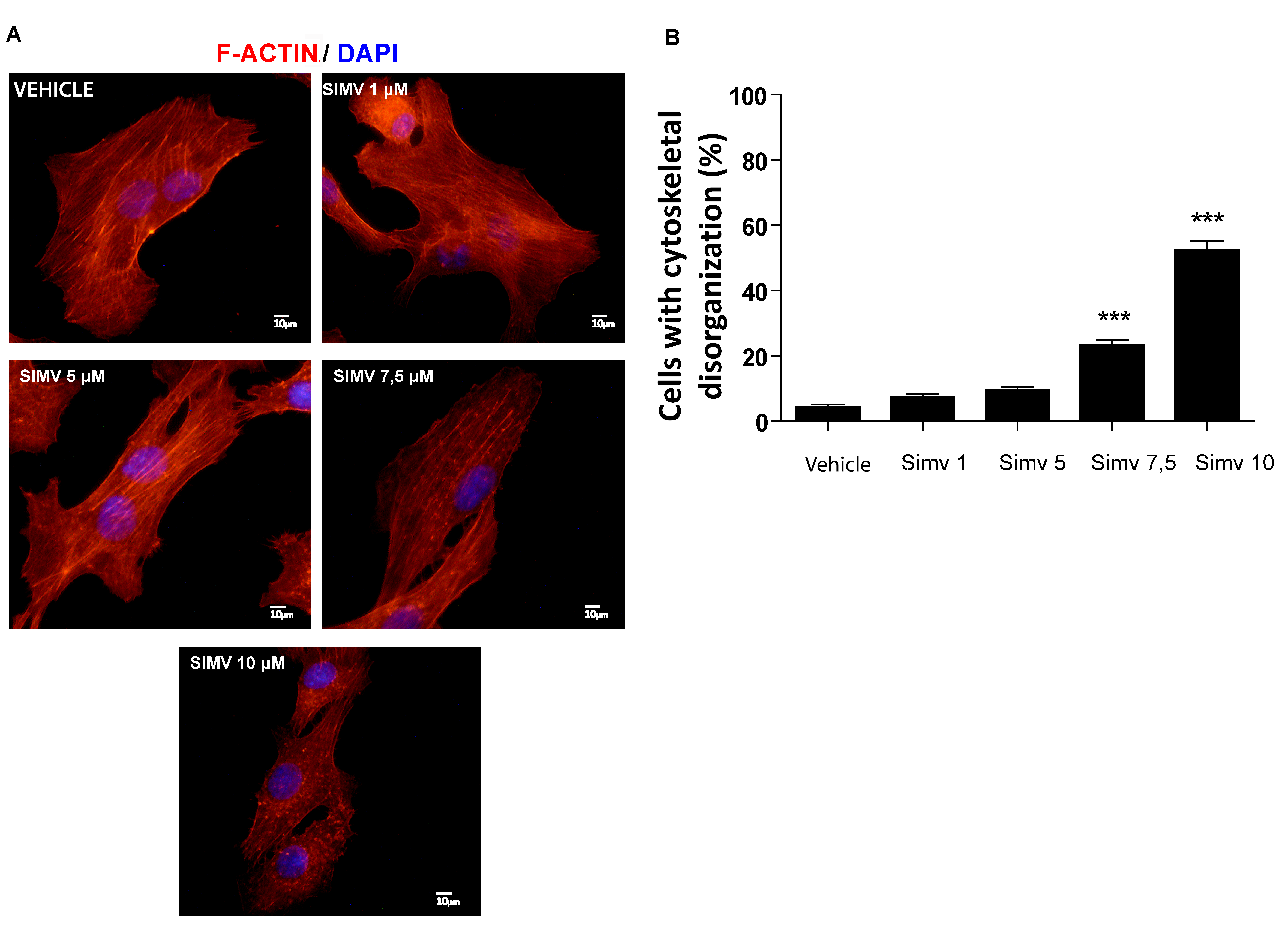

Supplement: S2 Fig — Cells were treated with vehicule (0.1% v/v DMSO) or simvastatin (1, 5, 7.5 and 10 μM) during 24 hours. A. Cells were fixed, permeabilized, stained with rhodamine-phalloidin and visualized by fluorescence microscopy (40X). B. Graphics analyses disrupted cytoskeleton cell percent. Data are shown as mean ± SD (n = 3 independent experiments). *** p<0,001 vs control. (TIF) [file pntd.0003770.s002.tif]

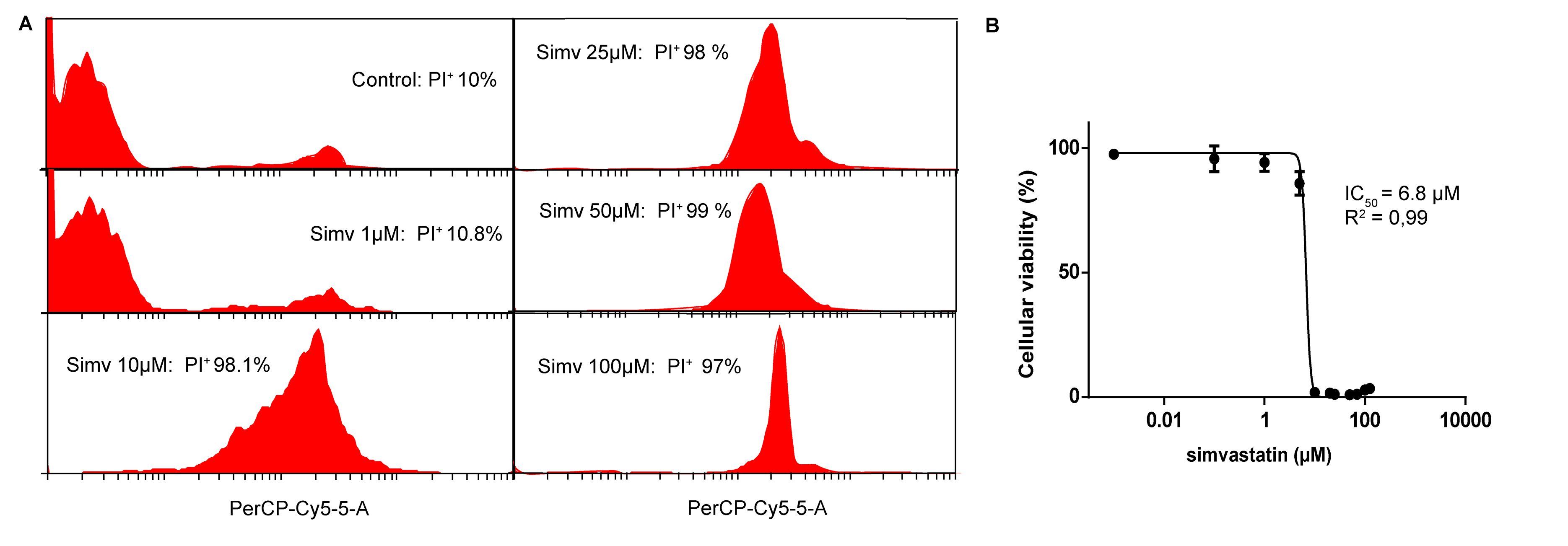

Supplement: S3 Fig — Cells were treated with vehicule control (0.1% v/v DMSO) or simvastatin (1, 5, 7.5 and 10 μM) during 24 hours. After 24hof incubation cells were stained with propidium iodide (0.5 μM) A. Representative histograms are shown for simvastatin-treated and control cells. Results are shown as the percentage of cells PI+. B. Graphs shown the histograms analyses. (TIF) [file pntd.0003770.s003.tif]

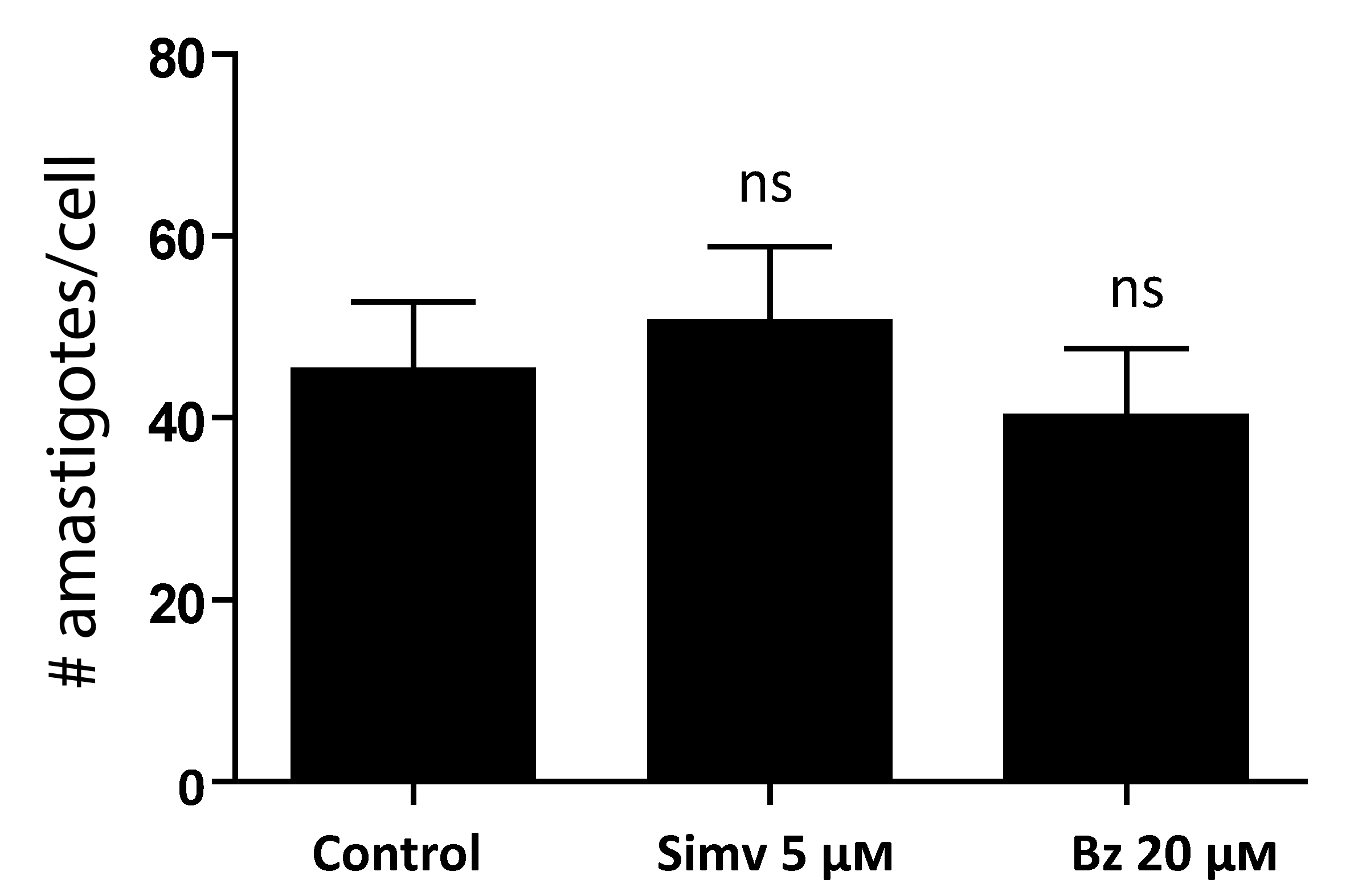

Supplement: S4 Fig — Endothelial-like (EA.hy926) and HUVEC cells were incubated with 5 μM simvastatin or 20 μM benznidazole 0.025% v/v final concentration. After 24 hours, the medium was replaced, and the cells were infected at a MOI of 10. After 16 hours of infection, cells were washed twice with fresh medium and the drug-free medium was changed daily. Then, after 72 hours of culture, the cells were fixed in cold methanol, and nuclei were stained with DAPI. The figure correspond to the quantitative comparison of the amastigote load for each experimental group. Each experimental condition was performed in duplicate and, for each duplicate, 10 photographs were taken. In average, there were counted 20.12±3.8 cells per photograph. Infected cells averaged 5.57±1.16 and healthy cells were 14.92±3.1. Infected to uninfected cells ration was 1:5. Controls were incubated with DMSO vehicle alone. The data are expressed as the mean ± SD from three independent experiments. NS: not significant after one-way ANOVA analysis. (TIF) [file pntd.0003770.s004.tif]
